# Stratification Modelling of Key Bacterial Taxa Driven by Metabolic Dynamics in Meromictic Lakes

**DOI:** 10.1038/s41598-018-27973-2

**Published:** 2018-06-22

**Authors:** Kaicheng Zhu, Federico M. Lauro, Haibin Su

**Affiliations:** 1 Institute of Advanced Studies, Nanyang Technological University, Singapore, Republic of Singapore; 2 Interdisciplinary Graduate School, Nanyang Technological University, Singapore, Republic of Singapore; 3 Asian School of the Environment, Nanyang Technological University, Singapore, Republic of Singapore; 4 Singapore Centre for Environmental Life Sciences Engineering, Nanyang Technological University, Singapore, Republic of Singapore; 5 Department of Chemistry, The Hong Kong University of Science and Technology, Hong Kong, China

## Abstract

In meromictic lakes, the water column is stratified into distinguishable steady layers with different physico-chemical properties. The bottom portion, known as monimolimnion, has been studied for the functional stratification of microbial populations. Recent experiments have reported the profiles of bacterial and nutrient spatial distributions, but quantitative understanding is invoked to unravel the underlying mechanism of maintaining the discrete spatial organization. Here a reaction-diffusion model is developed to highlight the spatial pattern coupled with the light-driven metabolism of bacteria, which is resilient to a wide range of dynamical correlation between bacterial and nutrient species at the molecular level. Particularly, exact analytical solutions of the system are presented together with numerical results, in a good agreement with measurements in Ace lake and Rogoznica lake. Furthermore, one quantitative prediction is reported here on the dynamics of the seasonal stratification patterns in Ace lake. The active role played by the bacterial metabolism at microscale clearly shapes the biogeochemistry landscape of lake-wide ecology at macroscale.

## Introduction

**Figure 1:**
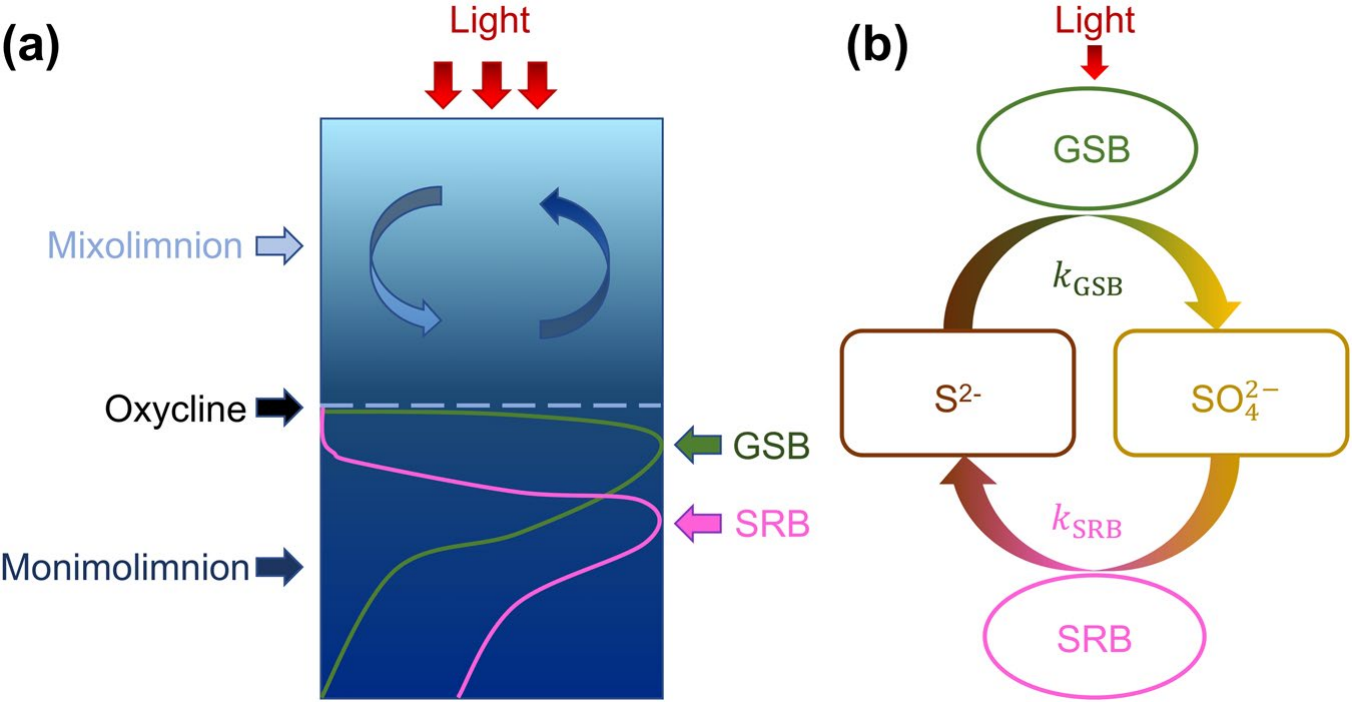
Schematic view of the meromictic lake system (**a**) and the sulfur cycle in the monimolimnion (**b**). The GSB and SRB take separate stratification layers at neighboring depths.

The taxonomic and functional stratification of microbial assemblages in meromictic lakes has been reported for more than a century^1–3^. The top portion of a meromictic lake, called the mixolimnion, usually extends only several meters below the lake surface. Here wind-driven circulation maintains active vertical mixing of the water column and because of the active lake-air exchange, the mixolimnion has elevated concentrations of dissolved oxygen supporting the diversity of aerobic biological systems. Conversely, in the monimolimnion, the anoxic bottom portion of a meromictic lake, many anaerobic bacterial species occupy discrete layers at different depths (Fig. 1a). Reduced sulfur compounds function as electron donors commonly among almost all phototrophic bacteria, especially the anoxygenic photosynthetic bacteria groups dominant in the monimolimnion^3,4^. Two bacterial phyla, the phototrophic sulfur bacteria and the sulfur-reducing bacteria (SRB), have been intensively studied because of their key roles in energy and nutrient cycling. The phototrophic sulfur bacteria consist of green sulfur bacteria (GSB) and purple sulfur bacteria (PSB) that share similar sulfur metabolism^4^. GSB and PSB conduct anoxygenic photosynthesis using sulfide (S^2−^) as a source of electrons and oxidizing it to sulfate $({{\rm{SO}}}_{4}^{2-})$, while SRB use ${{\rm{SO}}}_{4}^{2-}$ as an electron acceptor and reduce it to S^2−^, closing the sulfur cycle under anaerobic conditions. Despite the metabolic similarity between GSB and PSB, GSB have been reported to be the dominant sulfur bacterial species in some meromictic lakes^5–7^. While the detailed chemical and physical mechanisms behind these reactions are well understood, the details of the mutualistic relationship between the 2 major players in the sulfur cycle is still unclear^8^. Their stratification patterns are shown with the individual concentration profiles in meromictic lakes from polar to tropical regions^7,9,10^. Previous experiments have built a broad view of the distribution of microbial species as well as the nutrient species, but a detailed quantitative analysis has not been conducted, and a mathematical explanation of the causes determining their spatial distribution is still missing.

Here we establish a mathematical model based on the basic bacterium-nutrient metabolism and introduce the reaction-diffusion mechanism^11,12^ to explain the spatial distributions of both bacteria and nutrients. Our model focuses just on the sulfur-cycle-related biochemical species; it produces the stratification patterns with minimal complexity without the need for molecular biochemical details, which ensures that all quantities and functions involved in our model can be calculated analytically. The model covers the light supply factor of the anoxygenic photosynthesis in the sulfur cycle to illustrate the light-driven mechanism of the spatial pattern shifting. We apply both analytical and numerical methods and compare our simulated results with the experimental observations of two meromictic lakes from different biomes: Ace lake and Rogoznica lake^3,7,9^. The simplicity of this mathematical model provides an intuitive understanding of the macroscale spatial organization of the bacterial landscape depending on the microscale metabolic activity.

## Model

### Minimal Metabolic Reaction Model for Bacterium-Nutrient Dynamics

To simplify and generalize the cellular biochemical reactions, we build the bacterial metabolism based on just three key components: the nutrient consumption, the metabolic product generation, and the bacterial mortality. The consumption of nutrient involves the intake of extracellular substrate molecules and the biochemical conversion process, which provides energy and substrates for cell growth. With the nutrient being digested, metabolic end-products are generated and become substrates for the other species. During the growth driven by the resources supply, the bacterium may die due to aging or environmental pressures. We simplify the metabolism to involve only one type of nutrient and one type of end-product for each bacterial species, and the metabolic reactions are explicitly written in simple pathways: $B+N\rightleftharpoons B\cdot N\to \alpha B+W$, $B\to \varnothing$, and $B\cdot N\to \varnothing$, where *B* stands for the bacterium, *N* for the nutrient, and *W* for the waste. *B·N* is the complex of bacterium-nutrient binding, which corresponds to the digesting state; *B* shows the free state bacterium that is not in the nutrient consumption or the digestion process. The stoichiometric coefficient *α* is a growth parameter that shows the growing ratio of cell mass per digestion process, denoting the nutrient utilization rate. Since the digestion is a fast process during one cell cycle, we assume that fast equilibration of digestion-related reactions can be achieved, and relations between each molecular species are expressed as follows:

1 $$\frac{d[N]}{dt}{|}_{reaction}=-\,\frac{1}{\alpha -1}\{\frac{d{[B]}_{t}}{dt}{|}_{reaction}+{k}_{d}{[B]}_{t}\},$$

2 $$\frac{d[W]}{dt}{|}_{reaction}=-\,\frac{d[N]}{dt}{|}_{reaction}.$$

where the brackets denote the concentration of each species, [*B*]_*t*_ stands for the sum of the bacterial concentrations in both free and digesting states, and *k*_*d*_ illustrates the mortality rate of the bacterium^13^. These two equations describe the relations between the dynamics of three individual species. Because there is no explicit chemical reaction rate parameter involved in the molecular level, these equations establish a scale-independent relation, which can be applied to all bacterium-nutrient dynamic systems. The only parameters required in Eqs (1) and (2) are the nutrient utilization rate alpha and the mortality rate of the bacterium.

### Reaction-Diffusion Theory of the Bacterium-Nutrient System

Preliminary mathematical model of the metabolic reactions is based on the mass action law under homogenous conditions. To solve the bacterial stratification observed in meromictic lakes, the diffusion term is added to the metabolism-based dynamics for an explicit spatial dependence. The general reaction-diffusion dynamics of partial differential equation (PDE) describes the additive nature of the reaction and diffusion events in simple mathematic equation:

3 $$\frac{\partial C(\mathop{x}\limits^{\rightharpoonup },t)}{\partial t}=D{\nabla }^{2}C(\mathop{x}\limits^{\rightharpoonup },t)+f(C),$$

where *C* denotes the concentration at the position $\mathop{x}\limits^{\rightharpoonup }$ and time *t*, the functional *f* stands for the reaction rate based on the local concentration, and *D* is the coefficient of self-diffusion driven by the concentration gradient. Previous applications of reaction-diffusion theory in bacterial systems have been mostly focused on microscale cellular motion with numerical simulations^11,12^, but in this work, we aim at building analytical model for large scale system with fundamental metabolic reactions. Combined with our minimal metabolic reaction model, the steady-state spatial correlations of the nutrient, bacterium, and waster concentrations are expressed in the following equations:

4 $${D}_{N}{\nabla }^{2}[N]=\frac{1}{\alpha -1}\{-{D}_{B}{\nabla }^{2}[B]+{k}_{d}[B]\},$$

5 $${D}_{W}{\nabla }^{2}[W]=\frac{1}{\alpha -1}\{{D}_{B}{\nabla }^{2}[B]-{k}_{d}[B]\}.$$

Three concentration functions, [*N*], [*B*], and [*W*], are linked by Eqs (4) and (5). As a result, the explicit solutions can be calculated with one more restriction. There is not any molecular biochemical reaction rate involved in this PDE system, similar with the Eqs (1) and (2). The simplicity of the theory without biochemical parameters reveals the spatial distribution correlations of bacterium and nutrient species from the cellular levels and does not require detailed quantitative measurements on the molecular levels. However, since Eqs (4) and (5) are based on the steady-state assumption, these relations describe the spatial patterns on macro timescales where the bacterium-nutrient system is in or near the equilibration.

## Results and Discussion

### Reaction-Diffusion Theory Solves the Spatial Pattern of Bacteria and Nutrients Distribution

From the coarse-grained pathways of the GSB-SRB sulfur cycle (Fig. 1b), the reaction terms in the PDEs reflect the rate of photosynthesis of GSB, the sulfate-reduction rate by SRB, and the mortality rate of individual bacterial populations. The anoxygenic photosynthesis of GSB requires a supply of photons, resulting in two different metabolic states of GSB: one active-photosynthesis state near the top of the monimolimnion and one inactive-photosynthesis state deeper in the lake where light is highly attenuated. With our minimal metabolic reaction model, the reaction rates of different species are related with simple mathematical relations and few parameters. The relations between steady-state concentration profiles of individual species are calculated under these two conditions. In the area where light supply can trigger the GSB photosynthesis, we have following relations between the concentration profiles of different nutrient and bacterial species:

6 $${D}_{{N}_{1}}{\nabla }^{2}[{N}_{1}]=\frac{1}{{\alpha }_{1}-1}\{-{D}_{{B}_{1}}{\nabla }^{2}[{B}_{1}]+{k}_{d1}[{B}_{1}]\}+\frac{1}{{\alpha }_{2}-1}\{{D}_{{B}_{2}}{\nabla }^{2}[{B}_{2}]-{k}_{d2}[{B}_{2}]\},$$

7 $${D}_{{N}_{2}}{\nabla }^{2}[{N}_{2}]=\frac{1}{{\alpha }_{1}-1}\{{D}_{{B}_{1}}{\nabla }^{2}[{B}_{1}]-{k}_{d1}[{B}_{1}]\}+\frac{1}{{\alpha }_{2}-1}\{-{D}_{{B}_{2}}{\nabla }^{2}[{B}_{2}]+{k}_{d2}[{B}_{2}]\},$$

where *B*_1_ denotes the symbol for GSB, *B*_2_ for SRB, *N*_1_ for *S*^2−^, and *N*_2_ for ${{\rm{SO}}}_{4}^{2-}$. On the other hand, if there is no photon due to the poor light supply or light attenuation, the correlation becomes following equations:

8 $${D}_{{N}_{2}}{\nabla }^{2}[{N}_{2}]=\frac{1}{{\alpha }_{2}-1}\{-{D}_{{B}_{2}}{\nabla }^{2}[{B}_{2}]+{k}_{d2}[{B}_{2}]\},$$

9 $${D}_{{N}_{1}}{\nabla }^{2}[{N}_{1}]=\frac{1}{{\alpha }_{2}-1}\{{D}_{{B}_{2}}{\nabla }^{2}[{B}_{2}]-{k}_{d2}[{B}_{2}]\},$$

where only SRB (*B*_2_) is biologically active in the sulfur-related reaction cycle. Between these two extreme conditions of infinite and no light supply, there exists an intermediate environment where the light intensity decreases but still strong enough to support the photosynthesis of GSB, where the activity of GSB metabolism is limited by the light attenuation rather than the sulfide supply.

Another key factor in this biological system is that the total available volume for microbial growth is limited, i.e., there are maximum values of the concentrations of each bacterial species. It is based on the biological intuition that every living cell needs to occupy certain spatial volume to maintain their basic metabolic growth. This biophysical assumption can be expressed as ${V}_{1}[{B}_{1}]+{V}_{2}[{B}_{2}]\le {V}_{max}$, where *V*_1_ and *V*_2_ denote the volumes occupied by every single cell of GSB and SRB respectively, and *V*_*max*_ denotes the maximal limitation of the living space. With this limitation assumption, the steady-state concentration profiles are solved from reaction-diffusion equations in the monimolimnion:

10 $${[{B}_{i}]}_{j}={C}_{ij2}\cdot {e}^{-{K}_{ij}x}+{C}_{ij1}\cdot {e}^{{K}_{ij}x}+{C}_{ij0},$$

11 $${[{N}_{i}]}_{j}={c}_{ij2}\cdot {e}^{-{k}_{ij}x}+{c}_{ij1}\cdot {e}^{{k}_{ij}x}+{c}_{ij1}{(x-{x}_{ij})}^{2}+{c}_{ij0},$$

where i = 1 or 2 denoting the different bacteria and nutrients, the integer j varies from 1 to 6 standing for the six independent solutions under different biophysical restrictions about light supply level and volume limitation (Supplementary Information), and the capitalized parameters *C*_*ij*_ and *K*_*ij*_ are associated with bacteria and the lower-case letters for nutrients. Most of the variables involved can be analytically calculated from the boundary conditions between the layers, so they are not free parameters but indeed depends on the metabolic rate constants and the biological system scales; the parameter values are different for each solution, which explains the quantitative specificity of diverse metabolic states. This feature gives the biological significance of these different solutions: each one solution stands for a special biophysical condition for bacterial metabolism. Since *K*_*ij*_, the exponential factors related with spatial pattern, are different for every solution layer, measurement of the depth-dependent *K* value can directly show which specific layer exists at that depth and help us to understand the local bacterial metabolic state (Table 1).

**Table 1:** The exponential factors in the expression of bacterial concentrations (Eq. 10).

*i*	${{\boldsymbol{K}}}_{{\bf{1}}{\boldsymbol{i}}}^{{\bf{2}}}$	${{\boldsymbol{K}}}_{{\bf{2}}{\boldsymbol{i}}}^{{\bf{2}}}$
1	$\frac{\frac{{k}_{d1}}{{\alpha }_{1}-1}+\frac{{k}_{d2}}{{\alpha }_{2}-1}\cdot \frac{{V}_{1}}{{V}_{2}}}{{D}_{N1}{A}_{1}+\frac{{D}_{B1}}{{\alpha }_{1}-1}+\frac{{D}_{B2}}{{\alpha }_{2}-1}\cdot \frac{{V}_{1}}{{V}_{2}}}$	$\frac{\frac{{k}_{d1}}{{\alpha }_{1}-1}+\frac{{k}_{d2}}{{\alpha }_{2}-1}\cdot \frac{{V}_{1}}{{V}_{2}}}{{D}_{N1}{A}_{1}+\frac{{D}_{B1}}{{\alpha }_{1}-1}+\frac{{D}_{B2}}{{\alpha }_{2}-1}\cdot \frac{{V}_{1}}{{V}_{2}}}$
2	0	0
3	${{k}_{l}}^{2}$	${{k}_{l}}^{2}$
4	$\frac{\frac{{k}_{d1}}{{\alpha }_{1}-1}\cdot {D}_{N2}{A}_{2}+\frac{{k}_{d2}}{{\alpha }_{2}-1}\cdot {D}_{N1}{A}_{1}}{{D}_{N1}{D}_{N2}{A}_{1}{A}_{2}+\frac{{D}_{B1}}{{\alpha }_{1}-1}\cdot {D}_{N2}{A}_{2}+\frac{{D}_{B2}}{{\alpha }_{2}-1}\cdot {D}_{N1}{A}_{1}}$	$\frac{\frac{{k}_{d1}}{{\alpha }_{1}-1}\cdot {D}_{N2}{A}_{2}+\frac{{k}_{d2}}{{\alpha }_{2}-1}\cdot {D}_{N1}{A}_{1}}{{D}_{N1}{D}_{N2}{A}_{1}{A}_{2}+\frac{{D}_{B1}}{{\alpha }_{1}-1}\cdot {D}_{N2}{A}_{2}+\frac{{D}_{B2}}{{\alpha }_{2}-1}\cdot {D}_{N1}{A}_{1}}$
5	$\frac{{k}_{d1}}{{D}_{B1}}$	$\frac{{k}_{d1}}{{D}_{B1}}$
6	$\frac{{k}_{d1}}{{D}_{B1}}$	$\frac{{k}_{d2}}{{D}_{B2}+({\alpha }_{2}-1){D}_{N2}{A}_{2}}$

The six independent solutions correspond to different biological constraints: (1) the saturation layer of GSB together with SRB where there is strong light intensity but poor nutrient supply; (2) the saturation layer of GSB where both light and nutrient supply is enough; (3) the saturation layer of GSB and SRB where the light intensity decays below the critical value supporting a GSB full saturation; (4) the unsaturated region where light intensity is strong enough to trigger photosynthesis; (5) the saturation layer of GSB and SRB without light supply; (6) the unsaturated area without light supply.

**Figure 2:**
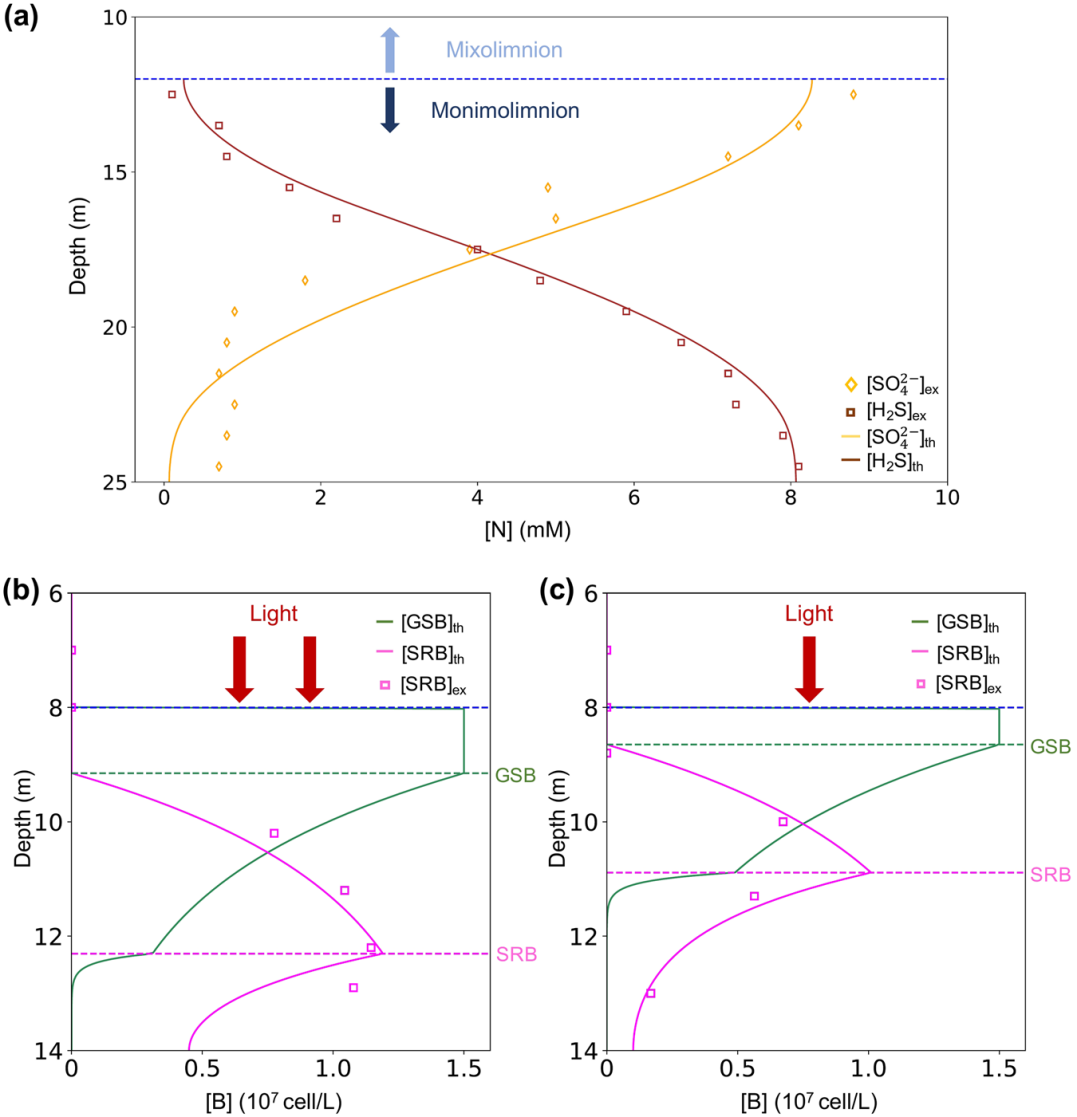
Concentration profiles of bacteria and nutrient species from theory and experiments. (**a**) The nutrient concentration profiles of Ace lake are fitted from reported data^3^. We plot the simulated seasonal dynamics of both GSB and SRB distributions in the summer (**b**) and the winter (**c**) based on the rRNA analysis of SRB in Rogoznica lake^9^. The analytical results of our model are presented by solid lines, and the experimental data are denoted by square/diamond shaped points. The blue dash line shows the position of the oxycline. The green dash lines and the violet dash lines denote the saturation depths of GSB and SRB respectively.

The ideal near-closed boundary conditions in the monimolimnion of a meromictic lake help to rule out three of all mathematically possible solutions, because they indicate net fluxes of nutrient across the system boundaries (Supplementary Information). If any of these three solutions exist, a net nutrient flux should be reported as sedimentation or molecule exchange with the mixolimnion. After eliminating the three solutions indicating an open system, we calculate the three possible concentration solutions that can exist in a closed system:

12 $$[{B}_{1}]=\{\begin{array}{ll}\frac{{V}_{max}}{{V}_{1}} & \,x < {x}_{s1}\\ \frac{{V}_{max}}{{V}_{1}}\cdot {e}^{-{k}_{l}(x-{x}_{s1})} & \,{x}_{s1}\le x < {x}_{s2}\\ \frac{{V}_{max}}{{V}_{1}}\cdot \frac{{e}^{-{k}_{l}({x}_{s2}-{x}_{s1})}}{{e}^{-{k}_{1}({x}_{s2}-2h)}+{e}^{{k}_{1}{x}_{s2}}}\cdot \{{e}^{-{k}_{1}(x-2h)}+{e}^{{k}_{1}x}\} & x\ge {x}_{s2}\end{array},$$

13 $$[{B}_{2}]=\{\begin{array}{ll}0 & x < {x}_{s1}\\ \frac{{V}_{max}}{{V}_{2}}\cdot \{1-{e}^{-{k}_{l}(x-{x}_{s1})}\} & {x}_{s1}\le x < {x}_{s2}\\ \frac{{V}_{max}}{{V}_{2}}\cdot \frac{1-{e}^{-{k}_{l}({x}_{s2}-{x}_{s1})}}{{e}^{-{k}_{2}({x}_{s2}-2h)}+{e}^{{k}_{2}{x}_{s2}}}\cdot \{{e}^{-{k}_{2}(x-2h)}+{e}^{{k}_{2}x}\} & x\ge {x}_{s2}\end{array},$$

where *k*_*l*_ is the light attenuation coefficient, ${k}_{1}=\sqrt{\frac{{k}_{d1}}{{D}_{B1}}}$, ${k}_{2}=\sqrt{\frac{{k}_{d2}}{{D}_{B2}+({\alpha }_{2}-1){D}_{N2}{A}_{2}}}$, *A*_2_ is the concentration ratio between the *N*_2_ and *B*_2_ in ideal exponential growth condition and *h* is the maximal depth of the monimolimnion from the chemocline. The three possible solutions demonstrate three layers of different biophysical conditions. In the chemocline-neighboring area, 0 < *x* < *x*_*s*1_, the biologically living space is fully occupied by GSB, forming the first saturation layer right below the top surface of the monimolimnion. Deeper into the lake, ${x}_{s1}\le x < {x}_{s2}$, SRB starts to take the dominating role of the microbial community by its concentration increase and the complementary decrease of GSB concentration. In this layer, the combination of two bacteria takes up all available biological volume to fill the space, building the second saturation layer. At the lower depth of the second saturation layer, SRB concentration reaches its maximum. Down to the very bottom of the lake, the concentrations of both bacterial species drop because of the lack of light, where no saturation of GSB or SRB is observed. The correlated nutrient concentrations are also analytically calculated (Supplementary Information) and compared with the data from Ace lake^3^. (Fig. 2a) The numerical fitting of the whole system involves only two degrees of freedom, the local concentrations of *S*^2−^ and ${{\rm{SO}}}_{4}^{2-}$ at the top surface of the monimolimnion: the boundary of $[{{\rm{SO}}}_{4}^{2-}]$ determines *x*_*s*1_ and *x*_*s*2_, thus fixing both bacterial profiles, while the boundary of $[{{\rm{S}}}^{2-}]$ determines the nutrient profiles. We also simulate the bacterial concentration profiles and quantitatively map the theoretical results with the observed seasonal dynamics of the stratification patterns in Rogoznica lake^9^. We find that the shifting of SRB saturation layer in Rogoznica lake is caused by the changes in the partition ratio of different sulfur species $({{\rm{S}}}^{2-}/{{\rm{SO}}}_{4}^{2-})$ which is determined by the light supply condition (Fig. 2b,c). Apart from this, our model predicts the GSB saturation layer thickness in Rogoznica lake by analyzing the SRB data, since *x*_*s*1_ and *x*_*s*2_ share the same degree of freedom. Our result suggests a highly saturated GSB layer thickness around 55 cm in the winter, which matches a separate experimental report^5^.

Because there are only two free parameters required for the system, the critical depths *x*_*s*1_ and *x*_*s*2_ depend on the nutrient constraint of [*N*_2_]_0_, the concentration of sulfate at the top surface of the monimolimnion. Both saturation layers increase their thicknesses, *x*_*s*1_ and $({x}_{s2}-{x}_{s1})$, with [*N*_2_]_0_ increasing (Supplementary Information). This mechanism sets up the restriction of [*N*_2_]_0_ for the stratification structure, since when *x*_*s*1_ drops to zero, the first saturation layer will not exist. If [*N*_2_]_0_ reaches an ultrahigh level, the entire of the monimolimnion will be occupied by the bacterial saturation layers.

### Light-driven Metabolic Mechanism Explains the Seasonal Stratification Shift

The reaction-diffusion model illustrates how the nutrient-based metabolisms shape the concentration profiles of both bacteria and nutrients species. In GSB the photosynthetic activity generates the energy supporting all the anabolic pathways and therefore growth, which has been reported to show a light dependence on top of nutrient conditions^14,15^. This biophysical principal then ensures the two degrees of freedom regarding the individual amounts of *S*^2−^ and ${{\rm{SO}}}_{4}^{2-}$ in the theory can be also interpreted as the combined sulfur amount and the systematic light supply level. The coarse-grained sulfur-cycle pathways (Fig. 1b) demonstrate this idea with a reversible biochemical reaction:

$${{\rm{S}}}^{2-}\rightleftharpoons {{\rm{SO}}}_{4}^{2-},$$

where the forward reaction rate is the GSB photosynthesis rate (*k*_*GSB*_) and the reverse reaction rate is the sulfur-reducing rate of SRB metabolism (*k*_*SRB*_). The reaction balance is achieved when ${k}_{GSB}\cdot [{{\rm{S}}}^{2-}]={k}_{SRB}\cdot [{{\rm{SO}}}_{4}^{2-}]$. Because of the complexity involved in the photon-excitation-driven sulfide oxidation and unknown details about SRB cellular pathways^14,15^, we propose a qualitative model to reveal the light-driven metabolic effect. We assume that the effective sulfate production rate from GSB photosynthesis can be simplified as

14 $${k}_{GSB}={k}_{ph}\cdot {e}^{I},$$

where *k*_*ph*_ is the prefactor with the unit of inverse time denoting the normalized photosynthesis rate at standard condition and *I* denotes the normalized light intensity. Since the total number of sulfur atoms is constant throughout a closed sulfur cycle, the light intensity changes the effective equilibrium constant between S^2−^ and ${{\rm{SO}}}_{4}^{2-}$ by tuning the value of *k*_*GSB*_ balanced with *k*_*SRB*_. The solutions of concentration profiles calculated from our theory show that the curvatures of nutrient concentration profiles are not affected by the integral values of each species. As a result, the light-driven reactions only affect the absolute concentration values of nutrient species but not their spatial patterns. When the light intensity grows, the amount of sulfate is increased by the higher photosynthetic activity, leading to larger *x*_*s*1_ and *x*_*s*2_ values, which indicates a downward shifting of both saturation layers. When the total amount of sulfur atoms is not conserved due to boundary leakage or sedimentation, the changes in the total sulfur amount may lead to similar effect of the change in light supply: when the system absorbs net sulfur flux from external supply, the required light intensity becomes lower to maintain the same saturation layers. This intuitive idea help us to understand the seasonal shifting of the SRB saturation layer depth in Rogoznica lake, where the net sulfur change is negligible within a single year: during the summer, there is a strong light supply at the lake surface promoting the anoxygenic photosynthesis and lowering the saturation layer depth and increasing the maximal concentration of SRB; in the winter, the sunlight decreases, so the saturation layer rises and the SRB maximal concentration drops. In addition, the model predicts an analogous shifting of saturation layers caused by the seasonal changes in light intensity and total sulfur storage in Ace lake by data analysis (Fig. S1). From the fitting of the nutrient data in Ace during 1990s^3^, we find that the unreported thickness of second saturation layer (*x*_*s*2_−*x*_*s*1_) was over 5 meters, but a recent metagenomics study in Ace lake has shown a thickness less than 2 meters^7^. The hidden shift of bacterial saturation layers indicates a light intensity decay, similar to the case from summer to winter in Rogoznica lake, or a sulfur loss in the monimolimnion. This correlation is also supported by similar shifting pattern of temperature and salinity profiles between Ace lake and Rogoznica lake^3,9^.

## Conclusions

In this study, a minimal reaction-diffusion model of the sulfur cycle driven by the metabolic activity of GSB-SRB is developed to explain the microbial stratification in meromictic lakes. Based on simplified light-driven bacterial metabolic dynamics, the model generates exact analytical results of distributions of all involved biological species. The bacterial layering patterns are calculated numerically using experimental parameters, and a quantitative bacterial distribution shift is predicted by comparison between theoretical solutions and experimental measurements. With an explicit mathematical expression, the model suggests that microbial metabolism plays an active role in shaping the landscape of ecological biogeochemistry.

## Electronic supplementary material

supplementary information
