# Supplementary material for: Stratification Modelling of Key Bacterial Taxa Driven by Metabolic Dynamics in Meromictic Lakes
Source: Sci Rep. 2018 Jun 22;8:9538. doi: 10.1038/s41598-018-27973-2 (PMC6015037; doi:10.1038/s41598-018-27973-2)
Supplement: Supplementary file 1 — supplementary information [file 41598_2018_27973_MOESM1_ESM.pdf]

Stratification Modelling of Key Bacterial Taxa Driven by Metabolic Dynamics in Meromictic Lakes

Kaicheng Zhu[1,2], Federico M. Lauro[3,4*], Haibin Su[1,5*]

[1] Institute of Advanced Studies, Nanyang Technological University, Singapore
[2] Interdisciplinary Graduate School, Nanyang Technological University, Singapore
[3] Asian School of the Environment, Nanyang Technological University, Singapore
[4] Singapore Centre for Environmental Life Sciences Engineering, Nanyang Technological University, Singapore
[5] Department of Chemistry, The Hong Kong University of Science and Technology, Hong Kong, China

Email: flauro@ntu.edu.sg; haibinsu@ust.hk

Derive the metabolic reaction relation between bacterium and nutrient

The reaction equations are shown below:

$$B + N \rightleftharpoons B \cdot N \rightarrow \alpha B + W,$$
$$B \rightarrow \emptyset,$$
$$B \cdot N \rightarrow \emptyset,$$

where B stands for the bacterium, N stands for the nutrient, and W stands for the waste, the product from B metabolism that cannot be used by itself. Parameter alpha shows the growth ratio per metabolic cycle, which corresponds to the nutrient utilization rate. After applying the mass action law of biochemistry, we derive the following equations for the metabolic rate dynamics.

$$\frac{d[N]}{dt}|_{reaction} = -k_f[B][N] + k_r[B \cdot N], \quad (S.1)$$
$$\frac{d[B]_t}{dt}|_{reaction} = \frac{d[B]}{dt} + \frac{d[B \cdot N]}{dt} = (\alpha - 1)k_{cat}[B \cdot N] - k_d[B]_t, \quad (S.2)$$
$$\frac{d[W]}{dt}|_{reaction} = k_{cat}[B \cdot N], \quad (S.3)$$

where the brackets denote the concentration of each species, k_f and k_r are the reaction rate constants for forward and backward eating process, k_{cat} is the catalytic reaction rate constant for the digestion process, and k_d shows the dying rate of the bacterial cell.

Assumption 1: Fast digestion equilibration

$$k_f[B][N] - (k_r + k_{cat})[B \cdot N] = 0 \quad (S.4)$$

To simplify the coupled dynamics of these molecular species, we apply our first assumption here that the digestion process reaches fast equilibrium compared with the dying process.

$$\frac{d[N]}{dt}|_{reaction} = -\frac{1}{\alpha-1}\{\frac{d[B]_t}{dt}|_{reaction} + k_d[B]_t\}, \quad (S.5)$$
$$\frac{d[W]}{dt}|_{reaction} = -\frac{d[N]}{dt}|_{reaction}. \quad (S.6)$$

Calculate the Concentration Profiles of Bacterial and Nutrient Species

The following are the PDEs of the reaction-diffusion system for the sulfur cycle:

$$\frac{\partial[N_1]}{\partial t} = D_{N_1}\nabla^2[N_1] + \frac{\partial[N_1]}{\partial t}|_{reaction},$$
$$\frac{\partial[B_1]}{\partial t} = D_{B_1}\nabla^2[B_1] + \frac{\partial[B_1]}{\partial t}|_{reaction},$$
$$\frac{\partial[N_2]}{\partial t} = D_{N_2}\nabla^2[N_2] + \frac{\partial[N_2]}{\partial t}|_{reaction},$$
$$\frac{\partial[B_2]}{\partial t} = D_{B_2}\nabla^2[B_2] + \frac{\partial[B_2]}{\partial t}|_{reaction}, \quad \text{(S.7)}$$

where $[B_1]$ is the concentration of GSB (the first bacterial species), $[B_2]$ is the concentration of SRB (the second bacterial species), $[N_1]$ is the concentration of the sulfide (the first nutrient species), and the $[N_2]$ is the concentration of sulfate (the second nutrient species). The four self-diffusion coefficients denote the diffusion constants of each species with corresponding subscripts.

The relations between steady-state concentration profiles of individual species are calculated under different light conditions. In the area where light supply can trigger photosynthesis by GSB, we have following relations between the concentration profiles of different nutrient and bacterial species:

$$D_{N_1}\nabla^2[N_1] = \frac{1}{\alpha_1-1}\{-D_{B_1}\nabla^2[B_1] + k_{d1}[B_1]\} + \frac{1}{\alpha_2-1}\{D_{B_2}\nabla^2[B_2] - k_{d2}[B_2]\},$$
$$D_{N_2}\nabla^2[N_2] = \frac{1}{\alpha_1-1}\{D_{B_1}\nabla^2[B_1] - k_{d1}[B_1]\} + \frac{1}{\alpha_2-1}\{-D_{B_2}\nabla^2[B_2] + k_{d2}[B_2]\}. \quad \text{(S.8)}$$

If there is no supply of photons, the correlations are described by the following equations:

$$D_{N_2}\nabla^2[N_2] = \frac{1}{\alpha_2-1}\{-D_{B_2}\nabla^2[B_2] + k_{d2}[B_2]\},$$
$$D_{N_1}\nabla^2[N_1] = \frac{1}{\alpha_2-1}\{D_{B_2}\nabla^2[B_2] - k_{d2}[B_2]\}, \quad \text{(S.9)}$$

where only SRB (B_2) is biologically active in the sulfur-related reaction cycle. Between these two extreme conditions of infinite and no light supply, there exists an intermediate state where the light intensity decreases but is still strong enough to support the photosynthesis of GSB, therefore the metabolic activity of GSB is limited by the light attenuation rather than the sulfide supply.

Assumption 2: $V_1[B_1] + V_2[B_2] \leq V_{max}$, where V_1 and V_2 denote the volumes occupied by every single cell of GSB and SRB respectively, and V_{max} denotes the maximal limitation of the living space.

Assumption 3: Due to the volume limitation of the assumption 2, the flux continuity of bacterial species does not hold at the boundary conditions where saturation layers meet the unsaturation layer. However, the continuity holds everywhere for nutrient species since there is no volume limitation for them.

Assumption 4: In free-space living condition where bacteria are not limited with the biological living space but metabolically active, the bacterial concentration is proportional to the nutrient concentration on the scales above colony level: $[N_1] = A_1[B_1]$, and $[N_2] = A_2[B_2]$.

Applying the above assumptions with the relation equations of the steady-state concentrations, we solve the reaction-diffusion equations analytically and get six

mathematical solutions. The detailed explicit forms of the six solutions are written as follows:

1. B_2 saturation layer with strong light supply but limited N_1 supply,

$$[B_1] = C'_{12} \cdot e^{-K_{11}x} + C'_{11} \cdot e^{K_{11}x} + C'_{10},$$
$$[B_2] = C''_{12} \cdot e^{-K_{21}x} + C''_{11} \cdot e^{K_{21}x} + C''_{10},$$
$$[N_1] = c'_{12} \cdot e^{-k_{11}x} + c'_{11} \cdot e^{k_{11}x} + c'_{10},$$
$$[N_2] = c''_{12} \cdot e^{-k_{21}x} + c''_{11} \cdot e^{k_{21}x} + c''_{10}, \qquad \text{(S.10)}$$

2. B_1 saturation layer with strong light supply,

$$[B_1] = C'_{20},$$
$$[B_2] = 0,$$
$$[N_1] = c'_{21} \cdot x^2 + c'_{20},$$
$$[N_2] = c''_{21} \cdot x^2 + c''_{20}, \qquad \text{(S.11)}$$

3. B_2 saturation layer with limited light supply,

$$[B_1] = C'_{31} \cdot e^{-k_l x} + C'_{30},$$
$$[B_2] = C''_{31} \cdot e^{-k_l x} + C''_{30},$$
$$[N_1] = c'_{32} \cdot e^{-k_l x} + c'_{31}(x - x_1)^2 + c'_{30},$$
$$[N_2] = c''_{32} \cdot e^{-k_l x} + c''_{31}(x - x_2)^2 + c''_{30}, \qquad \text{(S.12)}$$

4. Free-space with light supply,

$$[B_1] = C'_{42} \cdot e^{-K_{14}x} + C'_{41} \cdot e^{K_{14}x} + C'_{40},$$
$$[B_2] = C''_{42} \cdot e^{-K_{24}x} + C''_{41} \cdot e^{K_{24}x} + C''_{40},$$
$$[N_1] = c'_{42} \cdot e^{-k_{14}x} + c'_{41} \cdot e^{k_{14}x} + c'_{40},$$
$$[N_2] = c''_{42} \cdot e^{-k_{24}x} + c''_{41} \cdot e^{k_{24}x} + c''_{40}, \qquad \text{(S.13)}$$

5. B_2 saturation layer without light supply,

$$[B_1] = C'_{51} \cdot e^{-K_{15}x} + C'_{50} \cdot e^{K_{15}x},$$
$$[B_2] = C''_{52} \cdot e^{-K_{25}x} + C''_{51} \cdot e^{K_{25}x} + C''_{50},$$
$$[N_1] = c'_{53} \cdot e^{-k_{15}x} + c'_{52} \cdot e^{k_{15}x} + c'_{51}(x - x_3)^2 + c'_{50},$$
$$[N_2] = c''_{53} \cdot e^{-k_{25}x} + c''_{52} \cdot e^{k_{25}x} + c''_{51}(x - x_4)^2 + c''_{50}, \qquad \text{(S.14)}$$

6. Free space without light supply,

$$[B_1] = C'_{61} \cdot e^{-K_{16}x} + C'_{60} \cdot e^{K_{16}x},$$
$$[B_2] = C''_{61} \cdot e^{-K_{26}x} + C''_{60} \cdot e^{K_{26}x},$$
$$[N_1] = c'_{62} \cdot e^{-k_{16}x} + c'_{61} \cdot e^{k_{16}x} + c'_{60},$$
$$[N_2] = c''_{61} \cdot e^{-k_{26}x} + c''_{60} \cdot e^{k_{26}x}, \qquad \text{(S.15)}$$

where the exponential factors in bacterial concentration profiles are:

$$K_{11}{}^2 = K_{21}{}^2 = \left(\frac{k_{d1}}{\alpha_1 - 1} + \frac{k_{d2}}{\alpha_2 - 1} \cdot \frac{V_1}{V_2}\right) / \left(D_{N1}A_1 + \frac{D_{B1}}{\alpha_1 - 1} + \frac{D_{B2}}{\alpha_2 - 1} \cdot \frac{V_1}{V_2}\right),$$
$$K_{14}{}^2 = K_{24}{}^2 = \left(\frac{k_{d1}}{\alpha_1 - 1} \cdot D_{N2}A_2 + \frac{k_{d2}}{\alpha_2 - 1} \cdot D_{N1}A_1\right) / \left(D_{N1}D_{N2}A_1A_2 + \frac{D_{B1}}{\alpha_1 - 1} \cdot D_{N2}A_2 + \frac{D_{B2}}{\alpha_2 - 1} \cdot D_{N1}A_1\right),$$
$$K_{15}{}^2 = K_{25}{}^2 = K_{16}{}^2 = \frac{k_{d1}}{D_{B1}},$$
$$K_{26}{}^2 = \frac{k_{d2}}{\alpha_2 - 1} / \left(D_{N2}A_2 + \frac{D_{B2}}{\alpha_2 - 1}\right), \qquad \text{(S.16)}$$

After checking the continuity at boundaries, we find that the solution 1, 4, and 5 indicate net nutrient fluxes in or out of the whole system, which do not meet the requirement of an ideal closed system. Ruling out these three solutions, the other three solutions stand for three distinguishable biophysical layers are calculated with explicit expressions of parameters:

a. B_1 saturation layer with light supply, $0 \le x < x_{s1}$, (case 2)

$$[B_1] = \frac{V_{max}}{V_1},$$

$$[B_2] = 0,$$
$$[N_1] = \frac{1}{D_{N1}} \cdot \frac{k_{d1}}{\alpha_1 - 1} \cdot \frac{V_{max}}{V_1} \cdot x^2 + [N_1]_0,$$
$$[N_2] = -\frac{1}{D_{N2}} \cdot \frac{k_{d1}}{\alpha_1 - 1} \cdot \frac{V_{max}}{V_1} \cdot x^2 + [N_2]_0; \quad \text{(S.17)}$$

b. B_2 saturation layer with limited light supply, $x_{s1} \le x < x_{s2}$, (case 3)

$$[B_1] = \frac{V_{max}}{V_1} \cdot e^{-k_l(x - x_{s1})},$$
$$[B_2] = \frac{V_{max}}{V_2} \cdot \{1 - e^{-k_l(x - x_{s1})}\},$$
$$[N_1] = \frac{1}{D_{N1} {k_l}^2} \cdot \left\{\frac{k_{d1} - D_{B1} {k_l}^2}{\alpha_1 - 1} + \frac{k_{d2} - D_{B2} {k_l}^2}{\alpha_2 - 1} \cdot \frac{V_1}{V_2}\right\} \cdot \frac{V_{max}}{V_1} \cdot e^{-k_l(x - x_{s1})} - \frac{1}{2D_{N1}} \cdot \frac{k_{d2}}{\alpha_2 - 1} \cdot \frac{V_{max}}{V_2} (x - x_{N1})^2 + [N_1]_1,$$
$$[N_2] = -\frac{1}{D_{N2} {k_l}^2} \cdot \left\{\frac{k_{d1} - D_{B1} {k_l}^2}{\alpha_1 - 1} + \frac{k_{d2} - D_{B2} {k_l}^2}{\alpha_2 - 1} \cdot \frac{V_1}{V_2}\right\} \cdot \frac{V_{max}}{V_1} \cdot e^{-k_l(x - x_{s1})} + \frac{1}{2D_{N2}} \cdot \frac{k_{d2}}{\alpha_2 - 1} \cdot \frac{V_{max}}{V_2} (x - x_{N2})^2 + [N_2]_1; \quad \text{(S.18)}$$

c. Free space without light supply, $x_{s2} \le x \le h$, (case 6)

$$[B_1] = \frac{V_{max}}{V_1} \cdot \frac{e^{-k_l(x_{s2} - x_{s1})}}{e^{-k_1(x_{s2} - 2h)} + e^{k_1 x_{s2}}} \cdot \{e^{-k_1(x - 2h)} + e^{k_1 x}\},$$
$$[B_2] = \frac{V_{max}}{V_2} \cdot \frac{1 - e^{-k_l(x_{s2} - x_{s1})}}{e^{-k_2(x_{s2} - 2h)} + e^{k_2 x_{s2}}} \cdot \{e^{-k_2(x - 2h)} + e^{k_2 x}\},$$
$$[N_1] = -A_2 \frac{D_{N2}}{D_{N1}} \cdot \frac{V_{max}}{V_2} \cdot \frac{1 - e^{-k_l(x_{s2} - x_{s1})}}{e^{-k_2(x_{s2} - 2h)} + e^{k_2 x_{s2}}} \cdot \left\{e^{-k_2(x - 2h)} + e^{k_2 x}\right\} + [N_1]_2,$$
$$[N_2] = A_2 \frac{V_{max}}{V_2} \cdot \frac{1 - e^{-k_l(x_{s2} - x_{s1})}}{e^{-k_2(x_{s2} - 2h)} + e^{k_2 x_{s2}}} \cdot \{e^{-k_2(x - 2h)} + e^{k_2 x}\}. \quad \text{(S.19)}$$

From the boundary conditions we set, the parameters can be derived as:

$$x_{N1} = x_{N2} = \left(1 + 2\frac{k_{d1}}{\alpha_1 - 1} \cdot \frac{\alpha_2 - 1}{k_{d2}} \cdot \frac{V_2}{V_1}\right) x_{s1} + \frac{1}{k_l} \cdot \frac{\alpha_2 - 1}{k_{d2}} \cdot \frac{V_2}{V_1} \left\{\frac{k_{d1} - D_{B1} {k_l}^2}{\alpha_1 - 1} + \frac{k_{d2} - D_{B2} {k_l}^2}{\alpha_2 - 1} \cdot \frac{V_1}{V_2}\right\}, \quad \text{(S.20)}$$
$$[N_1]_2 = [N_1]_1 + \frac{D_{N2}}{D_{N1}} [N_2]_1, \quad \text{(S.21)}$$

where k_l is the light attenuation coefficient, $k_1 = \sqrt{\frac{k_{d1}}{D_{B1}}}$, $k_2 = \sqrt{\frac{k_{d2}}{D_{B2} + (\alpha_2 - 1) D_{N2} A_2}}$ and h is the maximal depth of the monimolimnion from the chemocline.

Parameter estimation and sensitivity analysis

Based on our minimal metabolic reaction model, the only microscale reaction rate parameter involved in the bacterium-nutrient correlation is the mortality rate, k_d. In the numerical analysis, we apply the reported values from real experiments in aqua environment for the dying rates of GSB and SRB[1-3]. For the maximum depth of the monimolimnion, h, we use the exact values of 13 meters for Ace lake and 6 meters for Rogoznica lake from lake measurements[4-6].

Other parameters are estimated without direct experimental supports, so we have conducted sensitivity analysis to test the robustness of the model. The parameters regarding the individual cellular volumes and the total available living space, V_1, V_2, and V_{max}, are normalized parameters that their exact values do not affect the distribution profiles but just determine the absolute concentration values of GSB and SRB; only the relative ratio between V_1 and V_2 determines the overall behaviors of the final solutions. Due to general experimental observations, we assume that GSB

and SRB have similar cell sizes, so $V_1 \approx V_2$. Besides, the parameter space consisting of other related parameters has been well examined. The bacterial distribution profiles show great robustness against variations in the ratio parameters A_1 and A_2, as well as the utilization rates $(\alpha_1 - 1)$ and $(\alpha_2 - 1)$: the saturation layer depths change within 1 meter of variation range while there are around three orders of magnitude of variance in A_1 and A_2, and two orders of magnitude of variance in $(\alpha_1 - 1)$ and $(\alpha_2 - 1)$. The numerical results also remain relatively stable as long as the diffusion constants, D_{B1}, D_{B2}, D_{N1}, and D_{N2}, vary within a fold change of 5.

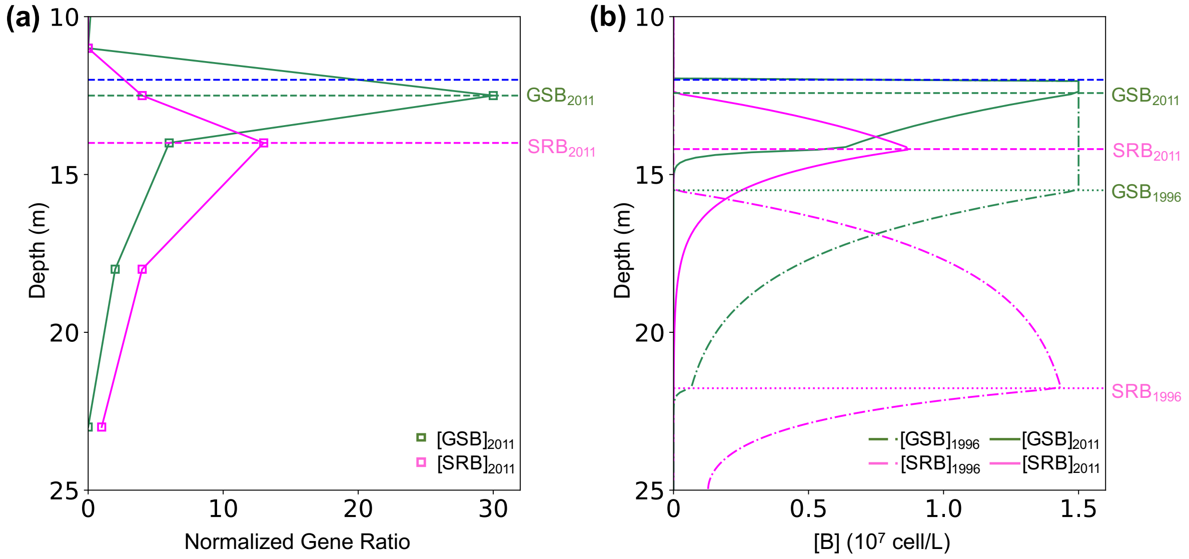

Supplementary Figure S1. Bacterial concentration profiles in Ace lake from experiment (a) and theory (b). In (a), we show the GSB and SRB concentration profiles of Ace lake in 2011 from experimental observations[4]. In (b), we plot the simulated results of bacterial distribution based on the nutrient data in 1996 and the bacteria data in 2011. A shift of bacterial saturation layers is predicted by our model. The blue dash line shows the position of the oxycline. The other horizontal lines denote the saturation depths of GSB and SRB at different time respectively.

References

1 Servais, P., Billen, G. & Rego, J. V. Rate of Bacterial Mortality in Aquatic Environments. *APPL ENVIRON MICROB* **49**, 1448-1454 (1985).

2 Biebl, H. & Pfennig, N. Growth yields of green sulfur bacteria in mixed cultures with sulfur and sulfate reducing bacteria. *ARCH MICROBIOL* **117**, 9-16, doi:10.1007/BF00689344 (1978).

3 Manske, A. K., Glaeser, J., Kuypers, M. M. M. & Overmann, J. Physiology and Phylogeny of Green Sulfur Bacteria Forming a Monospecific Phototrophic Assemblage at a Depth of 100 Meters in the Black Sea. *APPL ENVIRON MICROB* **71**, 8049-8060, doi:10.1128/AEM.71.12.8049-8060.2005 (2005).

4 Rankin, L. M., Gibson, J. A. E., Franzmann, P. D. & Burton, H. R. . The chemical stratification and microbial communities of Ace Lake, Antarctica: a review of the characteristics of a marine-derived meromictic lake. . *Polarforschung* **66**, 33-52 (1999).

5 Lauro, F. M. *et al.* An integrative study of a meromictic lake ecosystem in Antarctica. *ISME J* **5**, 879, doi:10.1038/ismej.2010.185 https://www.nature.com/articles/ismej2010185 - supplementary-information (2010).

6 Ciglenečki, I., Ljubešić, Z., Janeković, I. & Batistić, M. in *Ecology of Meromictic Lakes* (eds Ramesh D. Gulati, Egor S. Zadereev, & Andrei G. Degermendzhi) 125-154 (Springer International Publishing, 2017).
